# Effect of prolonged voluntary wheel running on oxidative stress and defence mechanisms in cortex and hippocampus of healthy female rats

**DOI:** 10.1113/EP092815

**Published:** 2025-05-31

**Authors:** Camilla Myrup Holst, Iria Esperon‐Abril, Frederik Bryske Juhl, Jesper Emil Jakobsgaard, Jonas B. Kristiansen, Kristian Vissing, Tinna Stevnsner

**Affiliations:** ^1^ Department of Molecular Biology and Genetics Aarhus University Aarhus Denmark; ^2^ Exercise Biology, Department of Public Health Aarhus University Aarhus Denmark

**Keywords:** antioxidant defence, brain, DNA repair, mitochondria, oxidative stress, physical exercise

## Abstract

Physical exercise promotes brain health and cognitive function possibly through mechanisms that include strengthened resistance to oxidative stress. However, limited research has explored the cumulative effects of regular voluntary exercise on both oxidative stress and defence mechanisms in hippocampus and cortex, two regions essential for cognitive function. Especially, adaptations in the young, healthy brain are insufficiently understood. This study investigates the impact of 8 weeks of voluntary wheel running on oxidative damage and counteracting defence mechanisms in the cortex and hippocampus of young, healthy female rats. To this end, we assessed oxidative damage to proteins and DNA, antioxidant defence, and DNA repair mechanisms, focusing on the base excision repair pathway. Our findings show that 8 weeks of voluntary exercise does not significantly modify oxidative damage or antioxidant defences in either cortical or hippocampal brain regions. Instead, the voluntary wheel running intervention led to a reduction in the levels of DNA polymerase β and mitochondrial apurinic/apyrimidinic endonuclease 1, key enzymes involved in base excision repair. Moreover, mitochondrial DNA copy number increased in the cortex, but decreased in the hippocampus, suggesting distinct regional adaptations. Collectively, these results indicate that the healthy young brain maintains redox homeostasis despite reduced DNA repair capacity. By analysing a comprehensive array of biomarkers in two brain regions, this study addresses gaps in our current knowledge on prolonged training and brain health and provides valuable insights into how regular exercise produces region‐specific and shared responses in the healthy state.

## Abstract (highlights)

- **What is the central question of this study?**
  There is limited understanding of how regular exercise simultaneously affects multiple measures of oxidative stress and defence mechanisms: how does prolonged voluntary exercise affect such measures in the young, healthy brain?
- **What is the main finding and its importance?**
  Eight weeks of voluntary running wheel exercise in female rats does not impact oxidative damage or antioxidant defence mechanisms in cortex and hippocampus, whereas key enzymes of DNA repair are reduced. These findings provide new detailed insights into the mechanisms of regular exercise in the young, healthy brain.

## 1 INTRODUCTION

The cortex and hippocampus represent two brain regions essential for cognitive function. The cortex governs executive functions, such as decision‐making, attention and problem solving, while the hippocampus is important for memory consolidation and learning (Ackerman, 1992). Deterioration in these brain areas can significantly impair their associated cognitive abilities. Consequently, understanding mechanisms and knowledge on strategies to counteract damage and improve cognitive function have great potential to ensure brain health.

Relative to its size, the brain is one of the most energy consuming organs in the mammalian body (Clarke and Sokoloff, 1999). To meet these energetic requirements, the brain is highly dependent on mitochondrial oxidative phosphorylation to ensure a constant production of energy in the form of ATP (Comyn et al., 2024). However, concomitantly with the production of ATP in the mitochondria, reactive oxygen species (ROS) are also produced. Under normal physiological conditions, ROS play important roles in cellular signalling and gene expression, whereas circumstances giving rise to excessive ROS can lead to oxidative stress and cause oxidative damage to nucleic acids and proteins, to negatively affect proteostasis, genome stability and mitochondrial function (Beckhauser et al., 2016). The brain is particularly vulnerable to oxidative stress (Cobley et al., 2018). Accordingly, oxidative stress has been shown to associate with decline in cognitive function with ageing and neurological disease (Ionescu‐Tucker & Cotman, 2021). ROS react with proteins to form oxidative modifications, of which protein carbonylation is considered one of the main and most dangerous changes due to its irreversible nature, and it is therefore often used as a marker of oxidative stress (Dalle‐Donne et al., 2003). Moreover, ROS can attack DNA, generating a wide range of lesions in the nuclear and mitochondrial genomes. Each mitochondrion contains several copies of the mitochondrial genome encoding proteins essential for the oxidative phosphorylation process. Due to the close proximity of the electron transport chain to the mitochondrial genome, the mitochondrial DNA (mtDNA) is prone to oxidative damage. This, in turn, can lead to mtDNA mutations and deletions, depletion of mtDNA copy number (mtDNAcn), and mitochondrial dysfunction (Gustafson et al., 2020). Consequently, mtDNAcn is considered a proxy measure of mitochondrial function and health (Castellani et al., 2020).

To cope with ROS and mitigate the resulting oxidative damage, defence and repair mechanisms have evolved. These rely on a first line of defence by antioxidant enzymes converting ROS into less reactive and harmful molecules, and a second line of defence by systems to handle ROS‐induced damage such as DNA repair mechanisms (Chatterjee & Walker, 2017). Among these, base excision repair (BER) is considered the primary mechanism for repairing oxidative DNA damage. In this regard, BER enzymes such as apurinic/apyrimidinic endonuclease 1 (APE1) and DNA polymerase β (POLB), which function under the upstream regulation of the neuronal growth factor brain‐derived neurotrophic factor (BDNF) (Lautrup et al., 2023), are considered central (Krokan & Bjørås, 2013).

Regular physical exercise can improve cognitive function, with most pronounced benefits reported during ageing and in neurological disease (Brown et al., 2013; Zhang et al., 2023). One of the mechanisms underlying the cognitive benefits may be an adaptational response that includes augmented defence and repair systems to lower oxidative stress within the brain (Ionescu‐Tucker & Cotman, 2021; Radak et al., 2016). Adaptational responses in cortex and hippocampus to regular aerobic exercise have been studied in aged rodents and rodent models of neurodegenerative disease, with findings indicating ameliorated resilience to oxidative stress in these brain regions (Bo et al., 2014; García‐Mesa et al., 2016; Lu et al., 2017; Marosi et al., 2012; Melo et al., 2019). However, regular exercise may benefit brain health not only in aged and sick populations; yet benefits and underlying molecular mechanisms in young, healthy populations remain understudied (Stillman et al., 2020). Regarding oxidative stress, a few studies have reported that, unlike findings in aged and sick rodents, regular exercise did not produce major changes in the oxidative balance in young adult male mice in cortex (Bartra et al., 2022) or in hippocampus (Bo et al., 2014) in response to regular, forced aerobic exercise. However, these studies assessed only a limited set of oxidative biomarkers, leaving a widespread analysis of oxidative damage and defence mechanisms unexplored. Given that the hippocampus and cortex regions of the young brain experience a high degree of brain plasticity (Larsen & Luna, 2018), their sensitivity and responses to exercise stimulation may be distinct from those observed at older age. Understanding exercise‐induced adaptations in the young brain, before onset of age‐related changes or disease, is crucial to establish a baseline and identify both universal and age‐ and disease‐specific molecular mechanisms. To this end, the main objective of our study was to provide a detailed, comparative characterization of exercise‐induced oxidative adaptations in response to regular voluntary exercise in two brain areas critical for cognitive function in the young, healthy brain. Specifically, we assessed the effects of an 8‐week voluntary wheel running protocol on oxidative stress and a broad range of molecular components of defence mechanisms in both cortex and hippocampus in healthy young female rats.

## 2 METHODS

### 2.1 Ethical approval

In brief, all procedures were approved by the Danish Animal Experimental Inspectorate (No. 2021‐15‐0201‐01028) and complied with Danish Animal welfare legislation and the Directive 2010/63/EU of the European Parliament and the Council of 22 September 2010. The experiments conformed to the principles and standards of reporting of animal experiments of *Experimental Physiology* (Grundy, 2015).

### 2.2 Animals and exercise intervention

The cohort of rats and the exercise intervention are described in Jakobsgaard et al. (2022). In brief, a total of 19 female wild‐type Wistar rats (Janvier Labs, Le Genest‐Saint‐Isle, France) were used. Upon arrival at 5 weeks of age, rats were acclimatized for 1 week in the animal facility. All housing comprised a thermostatically controlled environment at 21°C room temperature, with a 12/12 h light–dark cycle and ad libitum access to food and water. Following acclimatization, 6‐week‐old rats were randomized to 8 weeks of either running (runner, *n* = 9) or a non‐running intervention (sedentary, *n* = 10). The running training protocol was conducted as voluntary wheel running as previously described (Broch‐Lips et al., 2011). Rats allocated to the running group were housed individually in a cage with ad libitum access to a 35 cm‐diameter unloaded running wheel (Tecniplast, Buguggiate, Italy). Rats allocated to the sedentary group were similarly housed individually but without access to a running wheel. Running wheels were equipped with a magnet‐based odometer (BC 9.16; Sigma‐Elektro GmbH, Neustadt, Germany) by which weekly running distance was registered. The training protocol included an evaluation of the weekly running distance following the initial 2 weeks of the intervention period to confirm a sufficient activity level of each individual rat. All rats continued for the remaining 6 weeks of the intervention. Rats were euthanized at the end of the 8‐week intervention period at 14 weeks of age by an intraperitoneal injection of sodium pentobarbital (200 mg/kg) containing 10% lidocaine. Running wheels were locked 24 h prior to killing.

### 2.3 Tissue collection

Immediately after killing the rats, the skull was opened, and the brain was carefully lifted out of the cranial cavity. Cortex and hippocampus were quickly dissected from the fresh brain tissue according to standard procedures (Aboghazleh et al., 2024). Briefly, after removing the brain from the skull, the olfactory bulb, cerebellum and brainstem were excised. The brain was bisected along the midline to separate the right and left hemispheres. The hippocampus was carefully dissected from each hemisphere. Subsequently, the cerebral cortex was carefully separated from the underlying subcortical structures. The dissected tissue was snap‐frozen on dry ice and stored at −70°C until further analysis.

### 2.4 Protein extraction from tissue

Brain tissues were homogenized in two volumes (w/v) ice‐cold buffer I (10 mM Tris–HCl pH 7.8, 200 mM KCl, 1% protease inhibitor cocktail III (Merck, Darmstadt, Germany, cat. no. 539134)) by 10 pestle strokes. Samples were sonicated on ice followed by addition of one volume of buffer II (10 mM Tris–HCl pH 7.8, 200 mM KCl, 2 mM EDTA, 40% glycerol, 0.2% NP‐40, 4 mM dithiothreitol (DTT), 1% protease inhibitor cocktail III). Samples were incubated for 2 h at 4°C with shaking followed by centrifugation at 16,000 *g* for 15 min at 4°C. The supernatants were dialysed in dialysis tubes (Fisher Scientific Inc., Roskilde, Denmark, cat. no. 132655, molecular mass cut‐off: 6–8 kDa) against a dialysis buffer (10% glycerol, 50 mM KCl, 25 mM HEPES–KOH pH 7.0, 2 mM EDTA, 2 mM DTT) for 2 h at 4°C. Protein concentration was determined by Bradford protein assay (Bio‐Rad Laboratories, Hercules, CA, USA, cat. no. 5000006) and samples were aliquoted and stored at −70°C until further analysis.

### 2.5 Isolation of mitochondria

Mitochondria were isolated from brain tissue as described elsewhere (Gredilla & Stevnsner, 2012). Protein concentration was determined by Bradford protein assay and mitochondria fractions were aliquoted and stored at −70°C until further analysis.

### 2.6 Immunoblotting analysis

Protein extracts were separated by SDS‐PAGE and transferred to a polyvinylidene difluoride (PVDF) membrane by dry transfer using the iBlot dry blotting system (Thermo Fisher Scientific, Waltham, MA, USA) followed by blocking in 5% w/v milk in TBS‐T (20 mM Tris‐HCl pH 8.0, 137 mM NaCl, 0.05% Tween) for 1 h at room temperature. Thereafter, the membrane was incubated with primary antibody overnight at 4°C. This was followed by washing in TBS‐T, incubation with secondary antibody for 1 h at room temperature, and additional washing in TBS‐T. Primary antibodies utilized were as follows: rabbit anti‐BDNF (Abcam, Cambridge, UK, cat. no. ab108319, 1:1000, RRID: AB_10862052), rabbit anti‐APE1 (Thermo Fisher Scientific, cat. no. PA5‐29157, 1:000, RRID: AB_2546633), mouse anti‐APE1 (Novus Biologicals, Littleton, CO, USA, cat. no. NB‐100‐116, 1:1000, RRID: AB_10080558), rabbit anti‐POLB (Abcam, cat. no. ab175197, 1:1000), rabbit anti‐Flap structure‐specific endonuclease 1 (FEN1) (Novus Biologicals, cat. no. NBP1‐84697, 1:1000, RRID: AB_11036988), rabbit anti‐8‐oxoguanine DNA glycosylase 1 (OGG1) (Novus Biologicals, cat. no. NB100‐106, 1:500, RRID: AB_10104097), mouse anti‐superoxide dismutase 1 (SOD1) (Santa Cruz Biotechnology, Dallas, TX, USA, cat. no. sc‐101523, 1:500, RRID: AB_2191632), mouse anti‐superoxide dismutase 2 (SOD2) (Santa Cruz Biotechnology, cat. no. sc‐137254, 1:200, RRID: AB_2191808), mouse anti‐catalase (CAT) (Santa Cruz Biotechnology, cat. no. sc‐271803, 1:500, RRID: AB_10708550), mouse anti‐heat shock protein 60 (HSP60) (Santa Cruz Biotechnology, cat. no. sc‐271215, 1:1000, RRID: AB_10607973), and mouse anti‐β‐actin (Sigma‐Aldrich, St Louis, MO, USA, cat. no. A2228, 1:10,000, RRID: 476697). Secondary antibodies utilized were as follows: sheep anti‐mouse IgG horseradish peroxidase (HRP) linked (GE Healthcare, Chicago, IL, USA, cat. no. NA931, 1:5000, RRID: AB_772210) and donkey anti‐rabbit IgG HRP linked (GE Healthcare, cat. no. NA934, 1:5000, RRID: AB_772260). All antibodies were diluted in 5% w/v milk in TBS‐T. Detection was performed with ECL Prime Western Blotting Detection Reagents (Cytiva, Marlborough, MA, USA, cat. no. RPN2232) and visualized on an Amersham Imager 600 (GE Healthcare). Band intensities were quantified by ImageQuant TL software (v. 8.2.0, RRID: SCR_018374). In some cases, membranes were stripped with Restore PLUS Western blot stripping buffer (Thermo Fisher Scientific, cat. no. 46430) and re‐probed with primary antibodies against other antigens. Membranes were re‐probed for β‐actin and HSP60 as a loading control for whole tissue extracts and mitochondrial extracts, respectively. Protein levels were normalized to the loading control. Where applicable, an internal standard was loaded in gels, and measurements were normalized to the standard to minimize gel‐to‐gel variations.

### 2.7 Protein carbonylation analysis

Brain tissues were homogenized in 10 volumes (w/v) ice‐cold RIPA buffer (150 mM NaCl, 1% Triton X‐100, 10 mM Tris pH 8.0, 0.5% sodium deoxycholate, 1% protease inhibitor cocktail III, 1% β‐mercaptoethanol) by 10 pestle strokes. Homogenates were incubated for 30 min on ice and centrifuged at 15,000 *g* for 30 min at 4°C. Supernatants were collected and protein concentration determined by Bradford protein assay. Protein carbonylation was analysed using the OxyBlot Protein Oxidation Detection Kit (Millipore, cat. no. S7150) according to the manufacturer's protocol. Briefly, 10 µg of protein extracts was derivatized by 2,4‐dinitrophenylhydrazine followed by separation on a Tris–glycine SDS‐PAGE (5% stacking, 12% separating). Subsequently, proteins were transferred to a PVDF membrane, and the membrane was blocked with 1% (w/v) bovine serum albumin (BSA) in PBS‐T (PBS + 0.05% Tween‐20) followed by overnight incubation with rabbit anti‐dinitrophenylhydrazone antibody (1:150) at 4°C. After washing in PBS‐T, the membrane was incubated with goat anti‐rabbit IgG HRP linked (1:300) for 1 h at room temperature. Antibodies were diluted in 1% (w/v) BSA in PBS‐T. After additional washing, chemiluminescence detection was performed. Subsequently, membranes were stripped and re‐probed for β‐actin as a loading control.

### 2.8 DNA damage analysis and mtDNA copy number

Damage analysis of nuclear and mtDNA was conducted by long‐range PCR as previously described (Furda et al., 2014) with minor modifications. Due to limited amount of material, 10 mg of brain tissue from each rat was pooled within experimental groups (*n* = 9 per group), and DNA was purified from the pooled tissue using the QIAmp DNA Mini Kit (Qiagen, Hilden, Germany, cat. no. 51304) according to manufacturer's protocol. The quality of the DNA was assessed by agarose gel analysis and spectrophotometry, and the concentration was precisely determined with the Quant‐iT PicoGreen dsDNA Assay Kit (Thermo Fisher Scientific, cat. no. P7589) following manufacturer's protocol. For analysis of mtDNA, the DNA template was digested with *Xho*I enzyme to eliminate supercoiling, which may otherwise interfere with the efficiency of the downstream PCR reaction (Chen et al., 2007). *Xho*I cuts outside the mitochondrial DNA regions amplified in this study. One nanogram per microlitre DNA was incubated with 1U/µL *Xho*I (New England BioLabs, Ipswich, MA, USA, cat. no. R0146S) in 1xrCutSmart buffer (New England BioLabs, cat. no. B6004) at 37°C for 15 min followed by heat inactivation at 80°C for 20 min. To include measurement of oxidized base lesions in the analysis, 0.75 ng/µL DNA template was incubated with formamidopyrimidine DNA glycosylase (Fpg) enzyme (New England BioLabs, cat. no. M0240) at a concentration of 45 U/mL and 50 U/mL for analysis of Fpg‐sensitive sites in mitochondrial and nuclear DNA, respectively. The incubation was carried out for 30 min at 37°C in 1× Fpg reaction buffer (50 mM NaCl, 10 mM Tris–HCl pH 7.8, 1 mM DTT) followed by heat inactivation at 60°C for 10 min. In long‐range PCR, a 13.4 kb fragment of the mitochondrial genome and 12.5 kb fragment of the transient receptor potential cation channel, subfamily M, member 2 (TRPM‐2) gene (also known as the clusterin gene) in the nuclear genome we amplified using the LongAmp *Taq* PCR Kit (New England BioLabs, cat. no. E5200). Amplification of a 211 bp fragment of the mtDNA and 101 bp fragment of the TRPM‐2 gene was conducted with the *Taq* PCR Kit (New England BioLabs, cat. no. E5000). PCR reaction conditions were optimized for each type of PCR to ensure that measurements were conducted within the linear range of the reaction. In all instances, a control sample containing 50% of the DNA template was included. Primer sequences and PCR cycling conditions are displayed in Table 1. Amplified PCR fragments were resolved on agarose gels stained with GelRed Nucleic Acid Stain (BioNordika, Herlev, Danmark, cat. no. 41003) and visualized on an Amersham Imager 600 (GE Healthcare). Band intensities were quantified by ImageQuant TL software (v. 8.2.0, RRID: SCR_018374). The intensity of the long PCR fragment was normalized to intensity of the corresponding short fragment, which is assumed to be independent of damage, to adjust for differences in the amount of mitochondrial and nuclear DNA, respectively. Lesion frequency per 10 kb was computed as previously described (Ayala‐Torres et al., 2000) using λ = −ln(*A*_D_/*A*_O_), where *A*_D_ and *A*_O_ represent the relative amplification of treated and untreated samples, respectively. Non‐Fpg‐digested DNA from the sedentary group served as the untreated sample. Fpg‐sensitive sites were calculated as the reduction in relative amplification following Fpg treatment. The intensity of the short mitochondrial fragment was set relative to the intensity of the short nuclear fragment as a measure of the mtDNAcn.

**TABLE 1:** Primers and PCR conditions.

Target region	Primer	Sequence (5′‐3′)	PCR cycling conditions
Mitochondrial genome			
13.4 kb fragment	Fwd Rev	AAA ATC CCC GCA AAC AAT GAC CAC CC GGC AAT TAA GAG TGG GAT GGT CGG TT	94°C 30 s, 18 × (94°C 30 s, 65°C 13 min), 65°C 10 min
211 bp fragment	Fwd Rev	CCT CCC ATT CAT TAT CGC CGC CCT TGC GTC TGG GTC TCC TAG TAG GTC TGG GAA	95°C 30 s, 20 × (95°C 15 s, 60°C 30 s, 68°C 30 s), 72°C 5 min
Nuclear genome (TRPM‐2/clusterin gene)			
12.5 kb fragment	Fwd Rev	AGA CGG GTG AGA CAG CTG CAC CTT TTC CGA GAG CAT CAA GTG CAG GCA TTA GAG	94°C 2 min, 30 × (94°C 30 s, 65°C 12 min), 65°C 10 min
101 bp fragment	Fwd Rev	GAA AGT CCG AAG GAG CCC AG CCA GGG GGT TTT CCT AGC TC	95°C 2 min, 30 × (95°C 15 s, 60°C 30 s, 68°C 45 s), 72°C 5 min

### 2.9 AP endonuclease activity assay

Preparation of a radiolabelled oligonucleotide containing an apurinic/apyrimidinic (AP) site analogue (tetrahydrofuran), lysis of mitochondria and AP endonuclease activity assay were performed as previously described (Gredilla & Stevnsner, 2012). The linear range of the assay was determined by addition of increasing amounts mitochondrial extract. Based on this titration, 4 µg of extract was selected. Activity was normalized to the level of HSP60 protein in the mitochondrial extracts as determined by immunoblotting.

### 2.10 Statistics

Data from the two experimental groups were expressed relative to the sedentary group mean. Normal distribution of data was tested by the Shapiro–Wilk test and visual inspection of Q‐Q plots. Statistical comparisons between two groups were performed by a two‐tailed Student's unpaired *t*‐test, with Welch's correction when variances were unequal, in normally distributed data, and by a two‐tailed Mann–Whitney *U*‐test for non‐normally distributed data with exact *P*‐values. Data are given as means ± standard deviation (SD) unless otherwise stated. Statistical analysis was performed using GraphPad Prism software (v. 10.4.0, GraphPad Software, Boston, MA, USA; RRID: SCR_002798).

## 3 RESULTS

### 3.1 Voluntary running training

**FIGURE 1:**
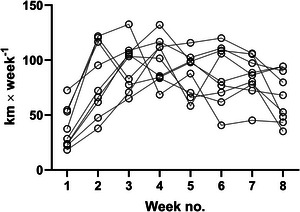
Running volume. Weekly running volume for each rat allocated to the voluntary running group (*n* = 9). Lines connect weekly data points related to a given rat.

Six‐week‐old female rats were randomly allocated to 8 weeks of voluntary wheel running intervention (runner, *n* = 9) or a non‐running intervention (sedentary, *n* = 10). Weekly running distance, body weight and composition characteristics of both groups are presented in Jakobsgaard et al. (2022). In brief, no differences were observed in body weight between the runner and sedentary group, while the weight of the heart and soleus muscles increased with exercise. The average running volume during the initial two intervention weeks was 118.6 ± 49.1 km. For the remaining 6 weeks, the average weekly running distance was 86.8 ± 13.6 km. Peak weekly running distance occurred between weeks 2 and 6 for all rats (Figure 1).

### 3.2 Oxidative damage and mitochondrial copy number

To investigate the effects of the prolonged training intervention on the maintenance of the young brain, we isolated cortex and hippocampus from the 14‐week‐old female rats after the voluntary wheel running intervention for 8 weeks.

**FIGURE 2:**
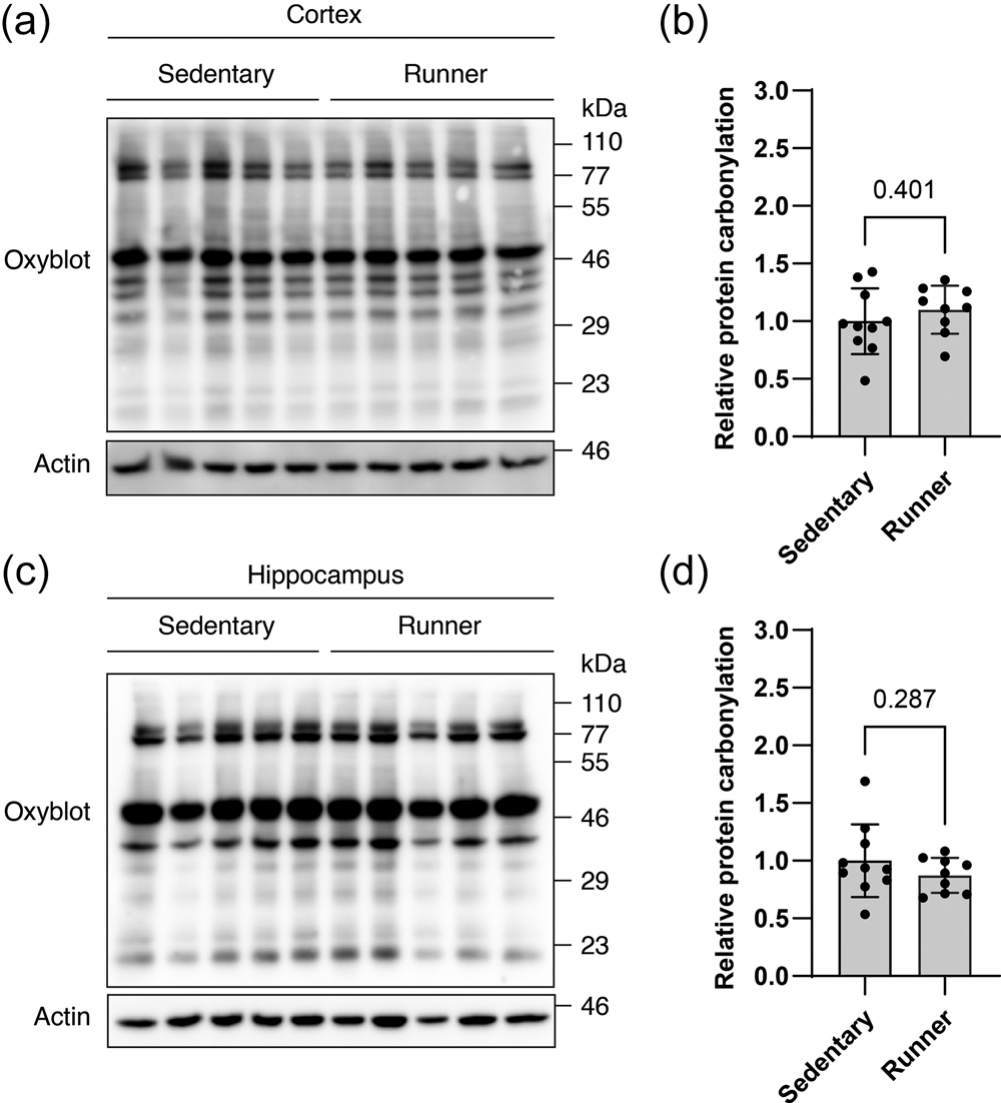
Protein oxidation. (a, c) Representative immunoblots of carbonylated proteins in whole tissue extract from cortex and hippocampus. (b, d) All values are normalized to β‐actin and set relative to the sedentary group mean. Bars represent means ± SD. *n *= 10 and 9 for sedentary and running group, respectively. Differences were evaluated by Student's unpaired *t*‐test.

To assess protein oxidation, protein carbonylation levels were measured in whole tissue extracts. The relative level of carbonylated proteins was similar between the sedentary group (*n* = 10) and the exercise group (*n* = 9) in cortex (1.00 ± 0.29 vs. 1.10 ± 0.21, *t*(17) = 0.862, *P *= 0.401, Student's unpaired *t*‐test, Figure 2a,b) and in hippocampus (1.00 ± 0.31 vs. 0.87 ± 0.15, *t*(17) = 1.099, *P *= 0.287, Student's unpaired *t*‐test, Figure 2c,d).

**FIGURE 3:**
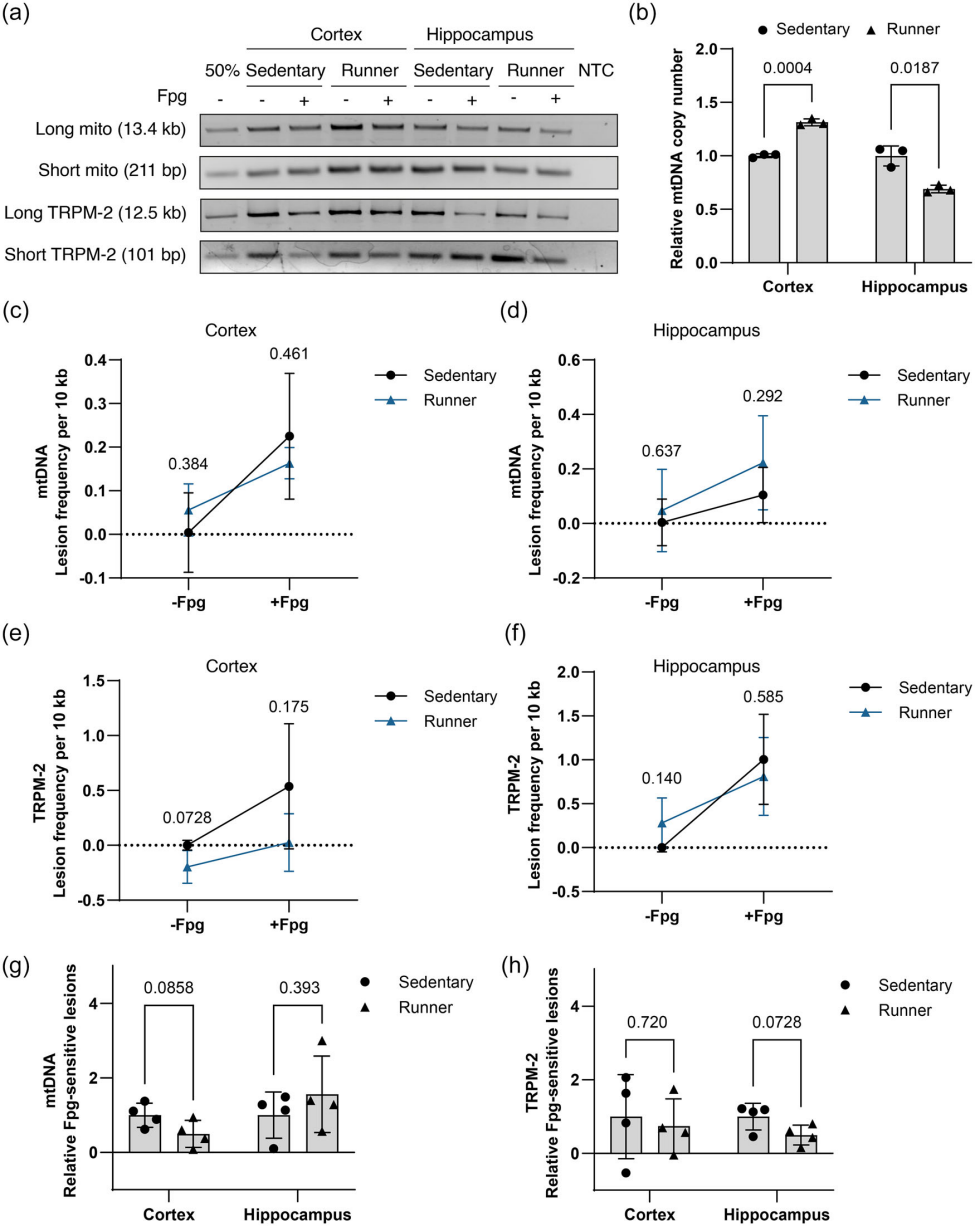
DNA damage and mtDNAcn. (a) Representative gels for regions analysed in the nuclear and mitochondrial DNA by long‐range PCR. 50%, control consisting of half the amount DNA template compared to the lane to the right (sedentary, cortex, −Fpg); NTC, no template control. (c–f) Values are expressed as lesion frequency/10 kb DNA and was calculated relative to sedentary group (−Fpg). (g, h) Relative Fpg‐sensitive lesions were calculated as the difference in relative amplification between non‐digested and Fpg‐digested DNA. (b, g, h) Values are set relative to mean of the sedentary group. Samples were pooled within experimental groups for the same tissue (*n* = 9 per group). Replicates represent repeated measurements of the pooled samples. Values represent means ± SD. Differences were evaluated by Student's unpaired *t*‐test with Welch's correction. mtDNAcn, mitochondrial DNA copy number.

To investigate the effect of the exercise protocol on DNA integrity, we assessed endogenous damage in the genome by long‐range PCR, which relies on an inverse correlation between the level of DNA damage and the PCR amplification efficiency. The analysis was conducted in two areas of the genome: within a large segment of the mitochondrial genome (13.4 kb out of 16.3 kb) and across a 12.5 kb segment of the TRMP‐2 gene in the nuclear genome. Both regions were normalized to a short fragment within the same region, for which amplification is assumed to be independent of damage (Figure 3a). To specifically examine oxidative damage, Fpg‐sensitive sites, which include oxidized purines and AP sites, were converted into single strand breaks by incubation with Fpg, thereby effectively stalling the polymerase during the PCR reaction. Furthermore, relative mtDNAcn was determined by setting amplification of the short mitochondrial fragment relative to the short nuclear fragment within the TRMP‐2 gene. For technical reasons, samples were pooled within experimental groups for these analyses (*n* = 9 pooled samples per group). mtDNAcn was increased by 31% in cortex (1.00 ± 0.02 vs. 1.31 ± 0.03, *t*(3.23) = 14.800, *P *= 0.0004, Welch's *t*‐test) and decreased by 31% in hippocampus (1.00 ± 0.10 vs. 0.69 ± 0.04, *t*(2.57) = 5.360, *P *= 0.0187, Welch's *t*‐test), demonstrating region‐specific effects of exercise on mtDNAcn (Figure 3b).

Analysis of relative mtDNA lesion frequency revealed no significant differences between the sedentary and runner group regardless of Fpg treatment in cortex (−Fpg; *t*(5.18) = 0.951, *P *= 0.384, Welch's *t*‐test, +Fpg; *t*(3.37) = 0.831, *P *= 0.461, Welch's *t*‐test, Figure 3c) and hippocampus (−Fpg; *t*(4.75) = 0.503, *P *= 0.637, Welch's *t*‐test, +Fpg; *t*(4.86) = 1.180, *P *= 0.292, Welch's *t*‐test, Figure 3d). Similarly, lesion frequency across the nuclear gene TRPM‐2 was not significantly altered by exercise in hippocampus (−Fpg; *t*(3.17) = 1.96, *P *= 0.140, Welch's *t*‐test, +Fpg; *t*(5.88) = 0.577, *P *= 0.585, Welch's *t*‐test, Figure 3f). Likewise, lesion frequency was unaltered in cortex (−Fpg; *t*(3.54) = 2.53, *P *= 0.0728, Welch's *t*‐test, +Fpg; *t*(4.22) = 1.63, *P *= 0.175, Welch's *t*‐test, Figure 3e). The relative level of Fpg‐sensitive sites, corresponding to the reduction in amplification upon Fpg digestion, showed no significant differences between the sedentary and runner group for mtDNA in hippocampus (1.00 ± 0.62 vs. 1.56 ± 1.03, *t*(4.93) = 0.935, *P *= 0.393, Welch's *t*‐test, Figure 3g) or cortex (1.00 ± 0.32 vs. 0.50 ± 0.36, *t*(5.92) = 2.06, *P *= 0.0858, Welch's *t*‐test, Figure 3g). Consistently, for the region within the nuclear gene TRPM‐2 there were no significant differences in Fpg‐sensitive sites in cortex (1.00 ± 1.14 vs. 0.74 ± 0.74, *t*(5.14) = 0.379, *P *= 0.720, Welch's *t*‐test, Figure 3h) or in hippocampus (1.00 ± 0.36 vs. 0.50 ± 0.27, *t*(5.57) = 2.21, *P *= 0.0728, Welch's *t*‐test, Figure 3h).

### 3.3 Defence mechanisms

**FIGURE 4:**
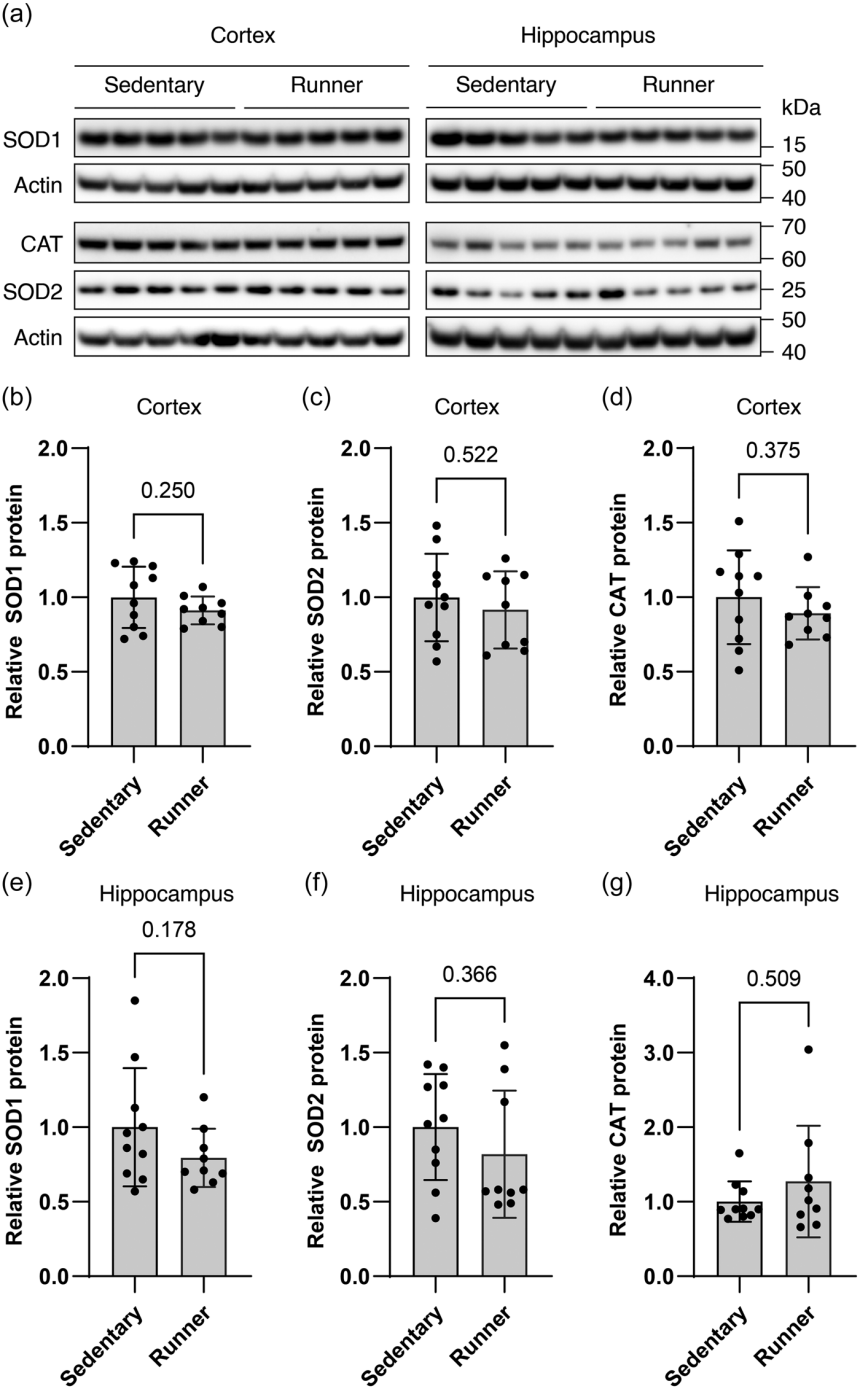
Antioxidant defence system. (a) Representative immunoblots of target antioxidant proteins in whole tissue extract from cortex and hippocampus. Proteins probed for are shown on the left. SOD1, superoxide dismutase 1; SOD2, superoxide dismutase 2; CAT, catalase. (b–g) All values are normalized to β‐actin and set relative to the sedentary group mean. Bars represent means ± SD. *n *= 10 and 9 for sedentary and running group, respectively. Differences were evaluated by Student's unpaired *t*‐test (c, d, e) with Welch's correction (b) or Mann‐Whitney *U*‐test (f, g).

We investigated the capacity of the cellular antioxidant defence system by measuring the protein levels of three key antioxidant enzymes in whole tissue extracts from the two brain regions from rats in the sedentary group (*n* = 10) and the exercise group (*n* = 9) (Figure 4a). In cortex, there were no differences in relative protein levels between the sedentary and the exercise group for the cytoplasmic superoxide dismutase 1 (SOD1) enzyme (1.00 ± 0.21 vs. 0.91 ± 0.09, *t*(12.87) = 1.204, *P *= 0.250, Welch's *t*‐test, Figure 4b), the mitochondrial SOD2 enzyme (1.00 ± 0.29 vs. 0.92 ± 0.26, *t*(17) = 0.654, *P *= 0.522, Student's unpaired *t*‐test, Figure 4c), or catalase (CAT; 1.00 ± 0.31 vs. 0.89 ± 0.17, *t*(17) = 0.911, *P *= 0.375, Student's unpaired *t*‐test, Figure 4d). Likewise, in hippocampus, relative protein levels were not significantly changed by exercise for SOD1 (1.00 ± 0.40 vs. 0.79 ± 0.20, *t*(17) = 1.405, *P *= 0.178, Student's unpaired *t*‐test, Figure 4e), SOD2 (1.00 ± 0.36 vs. 0.82 ± 0.43, *U *= 33.5, *P *= 0.366, Mann–Whitney *U*‐test, Figure 4f) and CAT (1.00 ± 0.27 vs. 1.27 ± 0.75, *U *= 36.5, *P *= 0.509, Mann–Whitney *U*‐test, Figure 4g).

**FIGURE 5:**
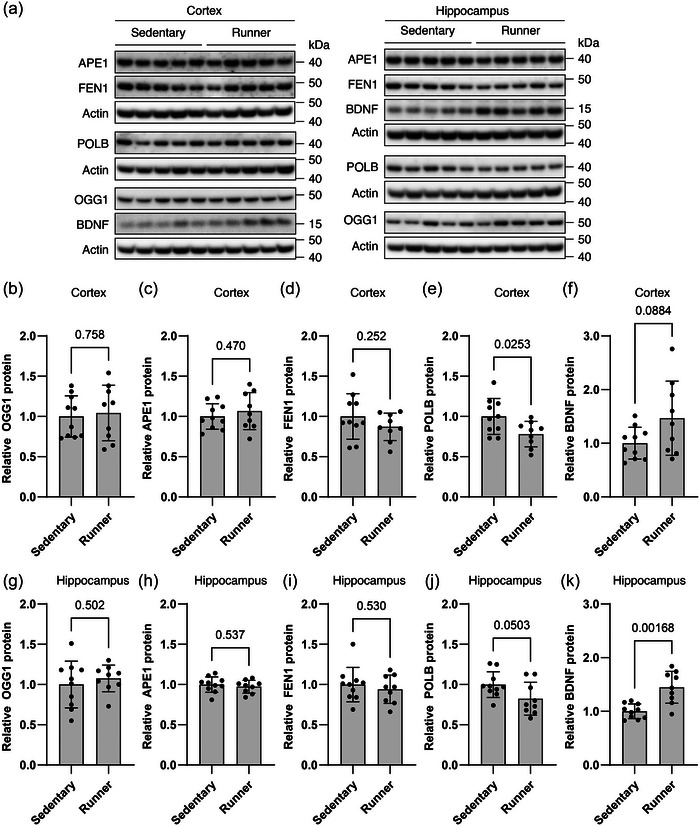
BER and neurotrophic factor. (a) Representative immunoblots of target proteins in whole tissue extract from cortex and hippocampus. Proteins probed for are shown on the left. OGG1, 8‐oxoguanine DNA glycosylase 1; APE1, apurinic/apyrimidinic endonuclease 1; FEN1, Flap endonuclease 1, POLB, DNA polymerase β; BDNF, brain‐derived neurotrophic factor. (b–j) All values are normalized to actin and set relative to the sedentary group mean. Bars represent means ± SD. *n *= 10 and 9 for sedentary and running group, respectively. Differences were evaluated by Student's unpaired *t*‐test (b–e, g–j) with Welch's correction (f, k). BER, base excision repair.

Similarly, we analysed the level of key enzymes involved in BER to evaluate the oxidative DNA damage repair capacity in the sedentary group (*n* = 10) and the exercise group (*n* = 9) (Figure 5a). Protein levels remained stable across experimental groups in cortex for 8‐oxoguanine DNA glycosylase 1 (OGG1; 1.00 ± 0.26 vs. 1.04 ± 0.34, *t*(17) = 0.314, *P *= 0.758, Student's unpaired *t*‐test, Figure 5b), APE1 (1.00 ± 0.16 vs. 1.07 ± 0.23, *t*(17) = 0.739, *P *= 0.470, Student's unpaired *t*‐test, Figure 5c) and Flap endonuclease 1 (FEN1; 1.00 ± 0.28 vs. 0.87 ± 0.17, *t*(17) = 1.185, *P *= 0.252, Student's unpaired *t*‐test, Figure 5d). The same phenomenon was observed in hippocampus for protein levels of OGG1 (1.00 ± 0.29 vs. 1.08 ± 0.17, *t*(17) = 0.685, *P *= 0.502, Student's unpaired *t*‐test, Figure 5g), APE1 (1.00 ± 0.10 vs. 0.97 ± 0.08, *t*(17) = 0.630, *P *= 0.537, Student's unpaired *t*‐test, Figure 5h) and FEN1 (1.00 ± 0.21 vs. 0.94 ± 0.18, *t*(17) = 0.640, *P *= 0.530, Student's unpaired *t*‐test, Figure 5i). POLB protein levels were significantly reduced with aerobic exercise by 22% (1.00 ± 0.22 vs. 0.78 ± 0.16, *t*(17) = 2.452, *P *= 0.0253, Student's unpaired *t*‐test, Figure 5e) in cortex and by 18% in hippocampus, with the latter nearly reaching statistical significance (1.00 ± 0.16 vs. 0.82 ± 0.20, *t*(17) = 2.107, *P *= 0.0503, Student's unpaired *t*‐test, Figure 5j).

In parallel, we measured the level of the neuronal growth factor BDNF. Exercise led to a 45% increase in hippocampal BDNF levels (1.00 ± 0.14 vs. 1.45 ± 0.30, *t*(11.0) = 4.13, *P *= 0.00168, Welch's *t*‐test, Figure 5k). In cortex, BDNF levels increased by a similar magnitude but with higher variability observed in this region, and the difference did not reach statistical significance (1.00 ± 0.30 vs. 1.47 ± 0.70, *t*(10.6) = 1.88, *P *= 0.0884, Welch's *t*‐test, Figure 5f). The high variability in cortical BDNF levels in the exercise group was not related to differences in total running distance over the 8‐week exercise intervention, as no statistically verifiable correlation was found between running distance and BDNF levels in cortex (Pearson *r *= 0.2709, *P *= 0.4804). On the other hand, despite showing lower variability, hippocampal BDNF levels displayed a positive correlation with total running distance (Pearson *r *= 0.6787, *P *= 0.0444).

**FIGURE 6:**
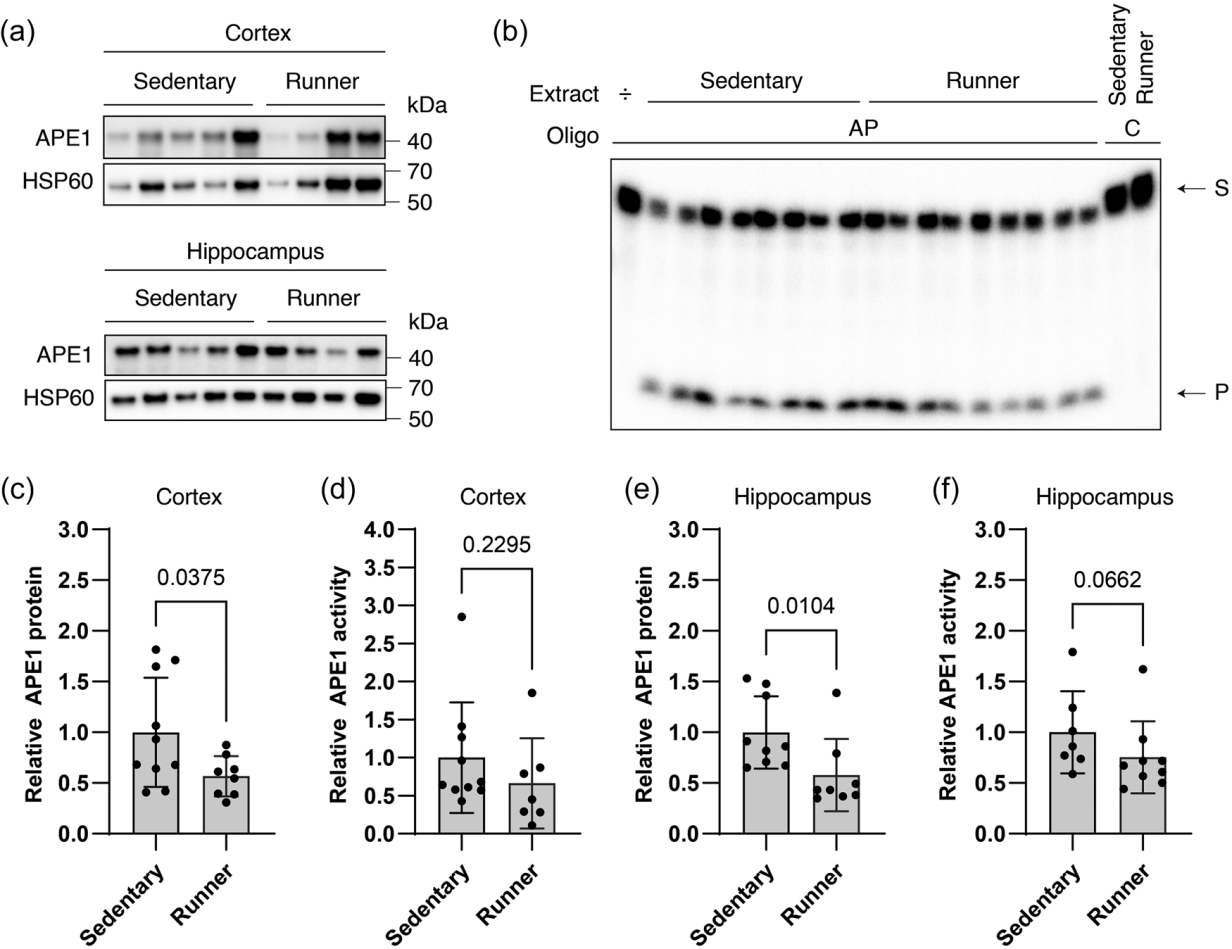
Mitochondrial BER. (a) Representative immunoblots of target proteins in mitochondrial extracts from cortex and hippocampus. Proteins probed for are shown on the left. APE1, apurinic/apyrimidinic endonuclease 1; HSP60, heat shock protein 60. (b) Representative gel from in vitro AP endonuclease activity assay with mitochondrial extracts from hippocampus. S, substrate; P, product; AP, oligonucleotide with AP site; C, control oligonucleotide without a lesion. (c–f) All values are normalized to HSP60 (mitochondrial loading control) and set relative to the sedentary group mean. Bars represent means ± SD. Differences were evaluated by Student's unpaired *t*‐test (c) or Mann–Whitney *U*‐test (d–f). (c) *n *= 10 and 8 for sedentary and running group, respectively. (d) *n *= 10 and 7 for sedentary and running group, respectively. (e) *n *= 9 and 8 for sedentary and running group, respectively. (f) *n *= 7 and 9 for sedentary and running group, respectively. One outlier was excluded from the analysis in the sedentary group for (e, f). Other discrepancies in sample size are due to technical issues in assays or insufficient yield from mitochondrial purification. BER, base excision repair.

To specifically investigate the impact of exercise on mitochondrial BER, we isolated mitochondrial fractions from both brain regions and evaluated the effect on APE1, a key BER enzyme that is readily detectable in mitochondria. The analysis of additional core BER enzymes was limited by the lack of functional antibodies with sufficient sensitivity for detecting enzymes present in low amounts and/or minimal material. In contrast to whole tissue, mitochondrial APE1 protein levels were significantly reduced in the exercise group with a reduction of 43% in cortex (1.00 ± 0.54 (*n* = 10) vs. 0.57 ± 0.20 (*n* = 8), *t*(11.87) = 2.342, Welch's *t*‐test, *P *= 0.0375) and 42% in hippocampus (1.00 ± 0.36 (*n* = 9) vs. 0.58 ± 0.36 (*n* = 8), *U *= 10, *P *= 0.0104, Mann–Whitney *U*‐test), showing similar effects across both regions (Figure 6c,e). Since the protein level of APE1 was decreased, we evaluated the consequence of this reduction by an in vitro AP endonuclease activity assay. Mitochondrial extracts were incubated with a double‐stranded oligonucleotide containing an AP site analogue, mainly recognized by APE1. Because of its endonuclease activity, APE1 converts this substrate into a single‐strand break, which can be recognized as a shorter product in a denaturing gel due to a radiolabel on the lesion‐containing strand (Figure 6b). Although the mean level of APE1 activity in cortex was around 34% lower in the group of runners compared to the sedentary group, no significant difference was present between the experimental groups (1.00 ± 0.73 (*n* = 10) vs. 0.66 ± 0.59 (*n* = 7), *U *= 22, *P *= 0.2295, Mann–Whitney *U*‐test, Figure 6d). Similarly, in hippocampus, the mean level APE1 activity was 25% lower, but did not reach statistical significance (1.00 ± 0.41 (*n* = 7) vs. 0.75 ± 0.36 (*n* = 9), *U *= 14, *P *= 0.0662, Mann–Whitney *U*‐test, Figure 6f).

## 4 DISCUSSION

In this study, we performed a widespread analysis on the oxidative–antioxidant balance in the cortex and hippocampus of young, healthy female rats exposed to 8 weeks of voluntary wheel running. The findings suggest that long‐term voluntary training does not significantly affect markers of oxidative damage to proteins and DNA, nor does it affect markers of the antioxidant defence system in the healthy brain. However, the exercise regimen led to a reduction in the level of POLB and mitochondrial APE1, key enzymes in the BER pathway, in both brain areas, along with region‐specific mitochondrial adaptations.

### 4.1 Oxidative damage and mitochondrial DNA copy number

The lack of significant differences in protein oxidation, oxidative DNA lesions or antioxidant enzymes between the sedentary and exercise group in either brain region suggests that prolonged voluntary exercise neither exacerbates nor alleviates oxidative damage nor modulates the antioxidant defence. This could indicate that the healthy, young brain harbours a naturally robust antioxidant defence system with an efficiency sufficient to maintain redox homeostasis without a need for enhancement, even under conditions of increased metabolic activity induced by exercise. As for protein oxidation, in opposition to the current findings, some interesting previous studies from the Radak laboratory on the effects of 8–15 weeks of aerobic exercise in healthy rodents have reported decreased protein oxidation in whole brain or hippocampus tissue in young, middle‐aged, and aged rats of either sex (Marosi et al., 2012; Ogonovszky et al., 2005; Radák et al., 2001). Noteworthy, all these studies utilize a forced treadmill model or swimming model combined with progressive overload training principles. The type and magnitude of stressors evoked by such an approach may differ from voluntary activity such as voluntary wheel running employed in our study. Since only modest decreases in protein carbonylation were reported in the aforementioned studies, the likely lesser intensity of voluntary wheel running might result in the absence of decreased protein oxidation. Moreover, in another study the same authors investigate middle‐aged inbred high capacity running male rats in which baseline protein oxidation seems unaffected and the effect of training on these animals is less clear (Sarga et al., 2013). Since the in‐breeding model is based on voluntary running animals, this together with the results of the current study could immediately support a contention that exercise intensity during training will dictate magnitudes of adaptational changes in protein oxidation. Despite reductions in protein carbonylation, the studies by the Radak group did not observe changes in DNA damage, indicated by 8‐oxoguanine (8‐oxoG) levels, or the DNA repair activity, evaluated as the activity toward 8‐oxoG lesions.

A striking observation of our study was the region‐dependent alteration in mtDNAcn following exercise, with an increase in cortex and a decrease in hippocampus. Only a few studies have previously reported on the brain tissue mtDNAcn in response to prolonged training. In one study, mtDNA increased in the cortex of aged male mice, whereas the authors did not measure hippocampal mtDNAcn (Lezi et al., 2014). In another study investigating effects of prolonged training in young adult male mice, no changes were observed in cortex and striatum, while once again, hippocampus was not included in the analysis (Herbst et al., 2015). The bidirectional response in our study may reflect distinct metabolic demands or regulatory mechanisms in the two brain regions. The observed increase in mtDNAcn in cortex could be interpreted as an adaptive response to support elevated energy demands. Conversely, a reduction in mtDNAcn may indicate a shift in mitochondrial dynamics such as increased mitophagy to enhance mitochondrial quality. Moreover, since some brain regions exhibit a heightened vulnerability to oxidative stress, including the hippocampus (Abbah et al., 2022; Candelario‐Jalil et al., 2001; Huang et al., 2012; Vinokurov et al., 2021; Wang & Michaelis, 2010), restricted mitochondrial function may serve as a protective mechanism to limit ROS production in these regions. Although decreased mtDNAcn is typically viewed as a negative outcome, a balanced, limited mitochondrial function may also be beneficial in certain contexts such as in vulnerable areas of the brain. Indeed, a controlled, chronic inhibition of mitochondrial respiration has been shown to reduce ROS production, improve the cellular energy homeostasis, and suppress age‐linked metabolic syndrome in ageing mice (Tavallaie et al., 2020). Moreover, citrate synthase activity and oxidative phosphorylation protein expression have previously been reported to remain unaltered in hippocampus after an extended period of voluntary wheel running, while cortex was not included in this analysis (Osburn et al., 2022). Other types of mitochondrial adaptations, such as altered mitochondrial crista density, have been observed upon prolonged training in skeletal muscle tissue (Groennebaek et al., 2020, 2018), and similar adaptational changes to training may also occur in brain tissue.

### 4.2 Defence and repair mechanisms

Compared to other studies on exercise effects on brain defence and repair mechanisms, our study includes a broader range of biomarkers in an attempt to provide for interpretations.

As for effects on defence mechanisms, this includes measurements on the antioxidant enzymes SOD1, SOD2 and CAT. None of these exhibited any changes in protein expression in either cortex or hippocampus in response to our training protocol. This is in immediate opposition to some other studies, reporting increases in antioxidant protein content or activity in brain tissue. Some of such studies were performed in rodent models of neurodegenerative disease, such as Parkinson's disease (Tuon et al., 2012) or Alzheimer's disease (Bo et al., 2014; García‐Mesa et al., 2016), which can be argued to constitute models of very different sensitivity to exercise and stressors and/or ability to perform exercise of an intended intensity. Findings of increased SOD or CAT protein expression with running training have also been reported in two studies on healthy rodents, with one reporting increase in cortex of young adult male mice (Bartra et al., 2022) and another in hippocampus of middle‐aged female rats (Marosi et al., 2012), but which both employed progressive intensity throughout running training and which may offer less familiarization to exercise stressor than voluntary running.

As for effects on components of DNA repair mechanisms, our exercise protocol only elicited subtle effects. While protein expression of most BER‐associated enzymes, including OGG1, APE1 and FEN1, remained unchanged, a significant reduction in POLB levels was detected in the cortex, and a similar observation was noted in the hippocampus. The observed reduction in mitochondrial APE1 levels, particularly in the hippocampus, suggests that exercise could modulate mitochondrial BER capacity possibly by regulating translocation of APE1 and other BER enzymes into mitochondria (Barchiesi et al., 2020; Prakash & Doublié, 2015). Only a few studies using a similar exercise intervention on a healthy rodent model have reported on exercise‐induced BER repair mechanisms in brain tissue and these are generally limited to analysis of fewer BER enzymes compared to the current study. In one such study by Yang et al. (2014), 10 consecutive days of voluntary wheel running in young adult male mice produced an increase in APE1 protein expression compared to non‐exercise control intervention. In another, by Vilela et al. (2017), 8 weeks of voluntary wheel running in aged male rats did not produce any changes in APE1 protein expression. The discrepant findings between the two studies could result from gradual familiarization with the exercise stress leading to gradually less necessity for APE1 for repair. This contention is supported by the fact that with a similarly prolonged exercise protocol as employed by Vilela et al., we observe similar lack of change not only in APE1 protein expression, but also OGG1 and FEN1 protein expression. Consistent with this, in a study by Bo et al. (2014), the level and activity of mitochondrial OGG1 was unaltered by 20 weeks of treadmill running in healthy, young male mice. The outright decrease in POLB protein expression in cortex as well as in mitochondrial APE1 immediately provide further support to the contention that exercise familiarization has occurred. In accordance with this, the absence of increased DNA damage indicates that the observed reduction in DNA repair capacity does not compromise genome stability under these experimental conditions. Instead, maintaining low levels of oxidative DNA damage as observed, even with a diminished repair capacity, may reflect an adaptive response. More specifically, a reduced demand for mitochondrial DNA repair could be attributed to enhanced mitochondrial quality control facilitating the removal of damaged and dysfunctional mitochondria (Kwon et al., 2021), thereby reducing the need for repair enzymes within this compartment. Accordingly, increased mitochondrial turnover may increase the mitochondrial efficiency, potentially lowering ROS production and the oxidative burden. Moreover, a reduction in the DNA repair capacity may represent a shift in cellular priorities, reallocating energy toward metabolism and synaptic plasticity rather than excessive maintenance functions. While the underlying biological basis of these findings is unclear, our study warrants further investigations into exercise‐induced changes in mitochondrial dynamics within the brain.

BDNF is an important player in brain health and has a crucial role in neuroplasticity and in promoting neuronal growth and survival by regulating various cellular processes. Importantly, our training model resulted in a significant increase in hippocampal BDNF levels, which were positively correlated with training volume. Cortical BDNF, despite greater variability, did not exhibit similar correlation to training volume. Exercise‐driven enhancement of neurotrophic support, reflected by elevated BDNF levels in the brain, is a well‐documented benefit of physical exercise (Berchtold et al., 2005; Cefis et al., 2023). Thereby, our findings align with previous studies, highlighting the role of exercise in promoting BDNF across ages and exercise regimens. We and others have previously shown that one of the ways BDNF protects neurons is by directly regulating expression of BER enzymes, including POLB and APE1, via the transcription factor CREB (Lautrup et al., 2023; Yang et al., 2014). Yang and colleagues have previously reported a simultaneous increase in BDNF and APE1 protein levels in cortex and hippocampus in 3‐ to 4‐month‐old male mice following a 10‐day voluntary running wheel exercise intervention (Yang et al., 2014). We did not observe an effect of the BDNF–BER axis in our exercise model, suggesting that this pathway is not engaged in healthy young female rats upon 8 weeks of voluntary wheel running. This is in contrast to the previously reported brief 10‐day intervention. In accordance with this, while acute responses to single‐bout exercise or brief exercise training periods may transiently stimulate this mechanism to counteract an exercise‐induced stress response, much higher total training volumes and training familiarization may not. Our study is also in accordance with the study by Vilela et al., in which an increase in BDNF levels in response to prolonged voluntary running was also not accompanied by an increase in APE1 protein expression (Vilela et al., 2017).

### 4.3 Limitations and future studies

Certain limitations should be considered when interpreting the findings. First, mitochondrial function was not directly assessed, leaving it uncertain how exercise‐induced changes in mtDNAcn may influence overall mitochondrial performance in this context. Furthermore, polarized cells such as neurons, exhibit a high degree of mitochondrial heterogeneity which may result in diverse responses in different pools of mitochondria (Gredilla et al., 2012; Pekkurnaz & Wang, 2022). Future studies could investigate the role of mitochondrial dynamics and function in more detail, to better understand the mechanisms underlying the region‐specific interplay between long‐term exercise and mitochondrial responses within the brain. Secondly, while the analysis of mtDNA provided robust coverage of over 80% of the mitochondrial genome, a limited region of the nuclear genome was examined. Given that DNA damage is not uniformly distributed across the genome (Amente et al., 2021; Poetsch, 2020), genome‐wide conclusions regarding nuclear DNA cannot be drawn. Thirdly, it should be acknowledged that examination of adaptations at the level of entire brain regions does not capture potential cell‐type‐specific responses of cell populations with varying vulnerabilities to oxidative stress or the influence of cellular crosstalk in defence (Lee et al., 2021; Wang & Michaelis, 2010). Fourth, we do not provide information on whether voluntary or forced exercise is more physiologically appropriate in in vivo animal exercise models. Whereas human individuals can be motivated to voluntarily increase intensity of exercise, it is less clear if voluntary wheel running leads animals to increase intensity rather than just exercise volume (running distance) per se. However, there are reports that indicate that adaptations in functional, physiological, biochemical and morphological features to voluntary activity resemble those acquired by forced activity in rats (Beleza et al., 2019). Moreover, the activity pattern of voluntary running in small rodents has been observed to be intermittent in nature, which could imply relatively high intensity intervals of running (Manzanares et al., 2018). On the other hand, forced exercise in animal models to ensure progression in exercise intensity may lead to a degree of stress that is not representative of normal physiological settings in small animals. Future studies on voluntary wheel running could attempt to monitor and report duration per distance, to allow indicatory information on whether intensity gradually increases with gradual increase in training status. Fifth, although our study found limited responses to prolonged exercise in the young brain, early‐life exercise may have long‐term effects on brain health (Greene et al., 2019). Further research is required to explore whether engaging in physical exercise during youth can positively impact brain health, including redox balance, in later life. Sixth, this study focused on female rats, while most previous studies have been conducted in male rodents. Discrepancies to past findings may reflect inherent biological differences between sexes, including hormonal influences. This underscores the need to better understand universal and sex‐specific responses in rodent studies (Barha et al., 2017). Finally, when extrapolating to human populations, it is important to consider differences in structure, function and complexity of the rodent and human brain, which may result in distinct responses (Wong et al., 2023).

### 4.4 Conclusions

In summary, by assessing the cumulative responses to prolonged training across multiple biomarkers of oxidative damage and defence, our study offers a comprehensive view of exercise‐induced modulation of the redox balance in the young brain with insights into region‐specific and shared adaptations in two brain regions crucial for cognitive function. Our findings demonstrate that the young, healthy brain adapts to regular voluntary training by stabilizing the magnitude of oxidative damage in the presence of unchanged antioxidant defences and reduced DNA repair capacity. Moreover, long‐term exercise is accompanied by distinct adaptations in mtDNAcn in the hippocampus and cortex suggesting region‐specific responses in mitochondrial dynamics. These findings offer a deeper understanding of the mechanisms underlying regular exercise in the young, healthy brain.

## AUTHOR CONTRIBUTIONS

Camilla Myrup Holst, Jesper Emil Jakobsgaard, Kristian Vissing and Tinna Stevnsner conceived and contributed to the design of the study. Jesper Emil Jakobsgaard performed the animal intervention, Camilla Myrup Holst isolated brain tissue, and Camilla Myrup Holst, Iria Esperon‐Abril and Frederik Bryske Juhl performed the subsequent experiments. Camilla Myrup Holst, Jonas Boritz Kristiansen, Iria Esperon‐Abril, Kristian Vissing and Tinna Stevnsner analysed and interpreted the data. Manuscript was written by Camilla Myrup Holst, Jonas Boritz Kristiansen, Iria Esperon‐Abril, Tinna Stevnsner and Kristian Vissing. All authors reviewed and approved the manuscript, and they agree to be accountable for all aspects of the work in ensuring that questions related to the accuracy or integrity of any part of the work are appropriately investigated and resolved. All persons designated as authors qualify for authorship, and all who qualify for authorship are listed.

## CONFLICT OF INTEREST

None declared.

## Data Availability

All data supporting the findings of the present study are available within the paper.
